# Comparative Analysis of Super-Shedder Strains of *Escherichia coli* O157:H7 Reveals Distinctive Genomic Features and a Strongly Aggregative Adherent Phenotype on Bovine Rectoanal Junction Squamous Epithelial Cells

**DOI:** 10.1371/journal.pone.0116743

**Published:** 2015-02-09

**Authors:** Rebecca Cote, Robab Katani, Matthew R. Moreau, Indira T. Kudva, Terrance M. Arthur, Chitrita DebRoy, Michael M. Mwangi, Istvan Albert, Juan Antonio Raygoza Garay, Lingling Li, Maria T. Brandl, Michelle Q. Carter, Vivek Kapur

**Affiliations:** 1 Department of Veterinary and Biomedical Science, The Pennsylvania State University, University Park, Pennsylvania, United States of America; 2 The Huck Institutes of the Life Sciences, The Pennsylvania State University, University Park, Pennsylvania, United States of America; 3 Food Safety and Enteric Pathogens Research Unit, National Animal Disease Center, Agricultural Research Service, U.S. Department of Agriculture, Ames, Iowa, United States of America; 4 Roman L. Hruska U.S. Meat Animal Research Center, Agricultural Research Service, U.S. Department of Agriculture, Clay Center, Nebraska, United States of America; 5 *E. coli* Reference Center, The Pennsylvania State University, University Park, Pennsylvania, United States of America; 6 Department of Biochemistry and Molecular Biology, The Pennsylvania State University, University Park, Pennsylvania, United States of America; 7 Produce Safety and Microbiology, Research Unit, Agriculture Research Service, U.S. Department of Agriculture, Albany, California, United States of America; University of Hyderabad, INDIA

## Abstract

Shiga toxin-producing *Escherichia coli* O157:H7 (O157) are significant foodborne pathogens and pose a serious threat to public health worldwide. The major reservoirs of O157 are asymptomatic cattle which harbor the organism in the terminal recto-anal junction (RAJ). Some colonized animals, referred to as “super-shedders” (SS), are known to shed O157 in exceptionally large numbers (>104 CFU/g of feces). Recent studies suggest that SS cattle play a major role in the prevalence and transmission of O157, but little is known about the molecular mechanisms associated with super-shedding. Whole genome sequence analysis of an SS O157 strain (SS17) revealed a genome of 5,523,849 bp chromosome with 5,430 open reading frames and two plasmids, pO157 and pSS17, of 94,645 bp and 37,446 bp, respectively. Comparative analyses showed that SS17 is clustered with spinach-associated O157 outbreak strains, and belongs to the lineage I/II, clade 8, D group, and genotype 1, a subgroup of O157 with predicted hyper-virulence. A large number of non-synonymous SNPs and other polymorphisms were identified in SS17 as compared with other O157 strains (EC4115, EDL933, Sakai, TW14359), including in key adherence- and virulence-related loci. Phenotypic analyses revealed a distinctive and strongly adherent aggregative phenotype of SS17 on bovine RAJ stratified squamous epithelial (RSE) cells that was conserved amongst other SS isolates. Molecular genetic and functional analyses of defined mutants of SS17 suggested that the strongly adherent aggregative phenotype amongst SS isolates is LEE-independent, and likely results from a novel mechanism. Taken together, our study provides a rational framework for investigating the molecular mechanisms associated with SS, and strong evidence that SS O157 isolates have distinctive features and use a LEE-independent mechanism for hyper-adherence to bovine rectal epithelial cells.

## Introduction

Shiga toxin-producing *Escherichia coli* (STEC) are major foodborne pathogens that cause significant morbidity and mortality with symptoms ranging from bloody diarrhea and hemolysis to the development of life-threatening hemolytic-uremic syndrome (HUS) [1–3]. STEC infections cause over 265,000 infections in the United States each year with *E. coli* O157:H7 (O157) accounting for close to a third of these illnesses [1, 3, 4]. Unfortunately, effective methods to control O157 colonization in cattle are lacking, resulting in a number of large, deadly multi-state outbreaks caused primarily through the consumption of contaminated food products (e.g. undercooked beef and fresh or processed produce), as well as through contact with infected animals and humans [4–8].

Cattle are the principal animal reservoir and carry O157 asymptomatically in their gastrointestinal tract, predominantly at the terminal recto-anal junction (RAJ). A subset of cattle, termed as “super-shedders”, is found to excrete O157 at extremely high levels (≥10^4^ CFU/g) in feces [9, 10]. Since most O157 strains are shed by cattle at 10 to 100 CFU/g of feces, super-shedders increase the potential of contaminating the surrounding environment and food supply by several orders of magnitude [1, 9, 10]. Colonization of the RAJ has been determined to be required for the high O157 shedding, a characteristic of super-shedders.

Mathematical modeling has revealed that the high transmission rate of O157 despite low pathogen prevalence among cattle is best explained by the presence of super-shedders. In fact, researchers in Scotland determined that high shedders account for 80% of the transmission events within herds even with the pathogen prevalent at rates less than 10% of animals [11]. Another epidemiological study of farms suggested that greater than 96% of O157 isolates originate from the less than 10% of animals that are super-shedders [12].

These higher transmission rates are caused by the increased likelihood of susceptible cattle coming in contact with high levels of bacteria, since as little as 300 CFU is sufficient to infect calves [13]. Thus, it is plausible that an animal excreting large numbers of O157 will pose a greater risk of spreading the pathogen to other cattle and into the food supply than the combined output of many animals that shed the pathogen at low levels [14, 15]. Mathematical modeling also suggests that the spread of O157 could be significantly reduced, if colonization in 5% of super-shedding animals was prevented [11].

Three principle components, or a combination thereof, likely contribute to the SS phenomenon: the microbe, the host, and the environment. Factors that may influence super-shedding at the microbe level may include strain-specific genomic characteristics such as the presence of unique virulence and/or adherence genes, strain lineage, genotype, or phage type, etc. Furthermore, strain variation in growth rates, biofilm formation, nutrient utilization, or the ability to survive and persist in the environment may also contribute to super-shedding [10, 13, 16–18]. Host factors that may play a role include the genotype, such as the presence of cellular or other receptors, as well as the innate and adaptive immune responses [9, 13]. Environmental factors that may influence the SS phenotype include external factors such as the season or climate, both of which are known to affect shedding levels, management practices (stocking densities, etc.) at the feedlot or pen where the animals are housed, in addition to internal factors such as host diet and the microflora in the cattle gastrointestinal tract [12, 16]. Understanding the roles of each of these components is important for the development of new methods to mitigate the high shedding and transmission of super-shedder isolates, and ultimately a reduction in food-borne illnesses associated with O157.

Localization in the terminal recto-anal junction (RAJ) is unique to O157 as other STEC serotypes are found in comparable levels throughout the large intestine [10, 19]. The RAJ is a unique region between the lymphoid follicle-associated columnar epithelial (FAE) cells found towards the distal colon and the stratified squamous epithelial (RSE) cells found towards the anal canal [10, 20, 21]. Experimental evidence has shown that swabbing the RAJ directly with O157 results in infection and carriage of the bacteria similar to cattle that have been naturally infected, indicating the importance of the RAJ in natural infections [22]. However, the molecular mechanisms that contribute to the adherence and colonization of O157, especially SS strains, to the bovine RAJ are unknown.

We describe here the complete genome sequence of SS17, a super-shedder strain of O157. Comparative analyses with O157:H7 strains Sakai, EDL933, EC4115, and TW14359 revealed polymorphisms at key loci encoding putative adherence and virulence factors, and a strikingly strong aggregative adherence pattern of SS isolates to bovine RSE cells that is LEE-independent. Together, our studies reveal strong evidence that SS isolates have a distinctive phenotype, and provide a rational framework to elucidate microbial factors and molecular mechanisms that contribute to super-shedding.

## Material and Methods

### Bacterial Strains

Super-shedder isolates of O157 strains were obtained and initially characterized at the USDA Meat Animal Research Center, Clay Center, NE. Fecal samples were collected by swabbing the RAJ from approximately 5,000 cattle during the summer months over a two-year period in U.S. Midwestern States. Samples were enumerated for O157 by plating onto CHROMAgar O157 (Becton Dickenson, Sparks, MD), and colony forming units (CFU) per swab were calculated as described previously [23]. Animals were classified as super-shedders when counts were equal or greater than 10^4^ CFU/swab [13, 17]. Up to twenty colonies were picked for PCR to confirm the genes for the O157 antigen, H7 flagella, γ-intimin, and at least one of the Shiga toxin genes [24]. Isolates were characterized by phage typing and *Xba*I-based pulse d-field gel electrophoresis (PFGE) as described [25]. This sampling produced 102 super-shedder isolates each representing a different animal. Supershedder isolate 17 (SS17) was chosen as a representative based on its genotypic and phenotypic characteristics that are also commonly found in other supershedder isolates and in strains that are likely to cause human illness. SS17 belongs to phage type 4 and was isolated at 3.1x10^5^ CFU/swab and has a closely related PFGE pattern [26]. *E. coli* O157 strains Sakai [NC_002695], EDL933 [NC_002655], TW14359 [NC_013008], and EC4115 [NC_011353] were used as reference genomes for comparative analyses [27–30]. In addition to EDL933, streptomycin-resistant derivative of strain 86–24 (86–24-Sm^R^; National Animal Disease Center (NADC), Ames, IA) was used as control O157 in the RSE cell adherence assay [31].

### Genomic DNA Extraction

Genomic DNA was isolated using QIAGEN DNeasy Blood and Tissue Kit (cat. no. 69504). Briefly, 1mL of overnight culture was centrifuged for 5min at 10,000xg in a microcentrifuge. The pellet was resuspended in provided buffer (ATL) and digested with 20μl proteinase K (20 mg/ml) for 30 mins at 56°C with a 5 sec vortexing halfway through the incubation. Cells were then processed as dictated in the protocol and eluted from the column with 100μl of ddH_2_O. DNA was quantified using Nanodrop (Thermo Scientific, Wilmington, DE) and aliquots stored at-20°C. Approximately 100ng of purified DNA was submitted for sequencing.

### Genome Sequencing and Assembly

Genomic DNA from SS17 was submitted to the Genomics Core Facility at The Pennsylvania State University for whole genome shotgun sequencing using the Ion Torrent PGM sequencer (Life Technologies, Grand Island, NY) [32]. Using both a 316 and a 318 sequencing chip, 4.2M reads with an average length of 165 ± 31 bases was obtained with 137.5 fold coverage. Both *de novo* and reference-guided assemblies were conducted to obtain large contigs. Genome assemblies were performed using DNASTAR SeqMan NGen 3.1 and Lasergene Suite (Madison, WI). A whole genome restriction optical map was generated using *BamH*I digestion by OpGen, Inc. (Gaithersburg, MD) to provide an isolate-specific reference on which to anchor the assembly [33]. The genome was closed using manual primer walking strategies and Sanger sequencing, and confirmation of ambiguous or low coverage areas was conducted using PCR and Sanger sequencing. The final assembly was compared to the optical map by generating an *in silico BamH*I restriction map of the sequence data.

### Annotation and Comparative Genomics

Initial automated annotation was performed using RAST (Rapid Annotation using Subsystem Technology; [34]) with manual annotation using Artemis [35] and BLASTp [36]. Whole genome alignments were performed using Mauve and the progressiveMauve algorithm to identify SNPs, genomic islands, and relative relationships between SS17 and reference strains [37, 38]. Lineage-specific polymorphism assay (LSPA) was performed using primers described in Yang, *et. al* [39] and Sanger sequencing to confirm insertions and deletions. Multilocus sequence typing (MLST) of SS17 and reference strains (Sakai, EDL933, EC4115, and TW14359) was performed using the MLST database at The University of Warwick (http://mlst.warwick.ac.uk/mlst/dbs/Ecoli; S1 Table). Further analysis of polymorphisms was conducted using a Perl script developed by Michael Mwangi to identify loci that were mutated in SS17 and not in reference genomes (S1 Program). Circular representations of the genome were generated with GenVision 10 (DNASTAR, Madison, WI) and the cladogram generated with FigTree v1.3.1 (Institute of Evolutionary Biology, University of Edinburgh, http://tree.bio.ed.ac.uk/).

The complete nucleotide sequence and annotation of SS17 have been deposited in GenBank with the following accession numbers: SS17 (CP008805), SS17 pO157 (CP008806), and SS17 pSS17 (CP008807).

### RSE Cell Adherence Assay

SS O157 and control non-SS-O157 strains were cultured overnight in Dulbecco Modified Eagle Medium-Low Glucose (DMEM; Gibco/lnvitrogen Corporation, Grand Island, NY) at 37°C without aeration, pelleted and resuspended in sterile saline as previously described [40]. RSE cells were suspended in 1 ml DMEM–No Glucose (DMEM-NG)±2.5% D+Mannose to a final concentration of 10^5^ cells/ml. For each strain, bacteria were mixed with RSE cells to achieve a final bacteria to cell ratio of 10:1. The mixture was incubated at 37°C with shaking for 4hrs. The mixture was then pelleted, washed thoroughly, and reconstituted to 100μl in dH_2_O. Drops of the suspension (2μl) were placed on Polysine (Thermo Scientific Pierce, Rockford, IL) slides and dried overnight under direct light to quench non-specific fluorescence, before fixing in cold 95% ethanol for 10 min. The slides were stained with 1% toluidine blue, or with fluorescence-tagged antibodies specific to the O157 antigen and cytokeratins of the RSE cells as previously described [20]. Adherence patterns on RSE cells were recorded as diffuse, aggregative, or non-adherent [40]. The percent of RSE cells with or without adhering bacteria was determined and recorded as strongly adherent when more than 50% of RSE cells had >10 bacteria attached, as moderately adherent when 50% or less of the RSE cells contained 1–10 adherent bacteria or as non-adherent when less than 50% of the RSE cells had only 1–5 adherent bacteria.

In order to determine involvement of the locus of enterocyte effacement (LEE)-encoded proteins, rabbit antisera generated against EspA, EspB, Tir, and Intimin (NADC Stocks), was pooled and tested at a 1:50 dilution [40]. Assays were preformed as described above with the antisera added to the resuspended bacterial pellets and incubated at 37°C for 30 min before mixing with the RSE cell suspension [40]. HEp-2 cells were also used in place of RSE cells for comparative purposes.

### Detection of Shiga Toxin (Stx)

ELISA plates were prepared by coating polystyrene microtiter plates with 100 µl of ceramide trihexoside (10µg/ml) (Matreya, LLC., Pleasant Gap, PA) in methanol. Wells were filled with 200 µl of blocking solution (4% BSA in 0.01 M PBS with 0.05% Tween 20) and the plates were incubated at 4°C with shaking overnight. The wells were washed twice with distilled water and stored at-20°C until use. *E. coli* cells were grown overnight in LB medium and 100 µl of cells were inoculated in fresh medium (5 mL). To induce cells, ciprofloxacin (10 mg/mL) was added and cells grown at 37°C for 6 hrs. The cells were centrifuged at 5,000x g for 3 min and the supernatant was used for the detection of Stx. Supernatant (200 µl) was dispensed in triplicate wells and incubated at 37°C with shaking for 1 hr and the wells were washed three times with 200 µl of distilled water. Primary antibodies for Stx1 or Stx2 (100 µl) (Santa Cruz Biotechnology, Inc) diluted (1µg/ml) in blocking solution were added to each well and the plates were incubated for 1 hr at 37°C. The wells were washed three times with 200 µl of distilled water and 100 µl of secondary antibody-peroxidase conjugate (1µg/ml) (Goat anti-mouse IgG, Thermo Scientific) prepared in blocking solution was added to each well and the plates were incubated for 1 hr at 37°C. After washing the wells three times with 200 µl of distilled water, 100 µl of 1-step ultra TMB (Thermo Scientific) was added to each well and the plates were incubated for 15 minutes at room temperature. An equal volume of stopping solution (1N HCl) was added and the optical density was read at 450 nm using a plate reader. Wells containing all components of the assay but without antigen, served as negative controls for determining the background. An OD450 of 0.2 above the background was considered positive for Stx.

### Single Step Inactivation of Genes

The genes *eae* and *eaeH* were deleted from SS17 using a combination of two-procedures previously described [41, 42]. First, kanamycin resistance (KanR) cassettes were generated with arms of homology to the gene of interest from the pACYC177 plasmid (New England BioLabs, Inc. Ipswich, MA) using PCR. Next, strains of SS17 were introduced with pKD119 (pBAD-λ RED) by electroporation, treated with arabinose, electroporated with the KanR cassette, and selected for using LB supplemented with 50μg/mL kanamycin. The confirmation of insertion and the location of the KanR cassette were confirmed by PCR and gel electrophoresis as well as sequencing at the Genomics Core Facility at The Pennsylvania State University (S2 Table).

## Results and Discussion

### SS17 Genome

**Figure 1:**
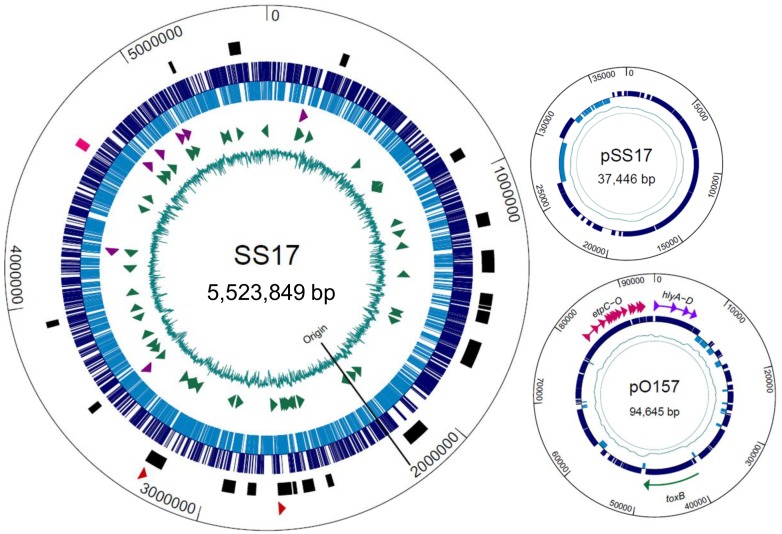
Genome map of SS17. Larger circle depicts chromosomal DNA with the smaller circles representing the two plasmids. **SS17**: From inner circle: percent GC (teal); tRNA (green arrowheads); rRNA (purple arrowheads); ORFs (forward direction-dark blue, reverse-light blue); location of phage (black bars) and LEE operons (pink); Shiga toxin II genes (red arrowheads); **pSS17**: percent GC (teal); ORFs (blue); **pO157**: percent GC (teal); ORFs (blue); hemolysin genes (purple), *toxB* gene (green) and *etp* operon (pink).

The chromosome of SS17 has a size of 5,523,849 bp and encodes for 5,430 open reading frames (Fig. 1, Table 1). Further analysis revealed 107 tRNAs and 22 rRNAs, which is consistent with other sequenced *E. coli* O157:H7 strains [27–30] (Fig. 1, Table 1). The SS17 genome contains two plasmids, pO157 (94,645 bp) and a smaller plasmid we termed pSS17 (37,446 bp) (Fig. 1). pO157 contains 111 genes with an average length of 744 bp, 87% coding, and a gene density of 1.172 genes per kb. Carried within the pO157 are genes for the production of hemolysin (*hlyABCD*), cytotoxin (*toxB*), the type V secretion serine protease (*espP*) and the type II secreted proteins (*etp* operon), which is highly similar to the large virulence plasmid found in O157 strains (Fig. 1). pSS17 is similar to the plasmid pEC4115 in the reference O157 strain EC4115 [27], and encodes a number of conjugal transfer, type IV secretion system, and hypothetical proteins.

**Table 1:** Genome statistics of SS17 and reference O157 strains.

	**SS17**	**EC4115**	**TW14359**	**Sakai**	**EDL933**
Length of Sequence (Mb)	5.52	5.57	5.52	5.49	5.53
G+C ratio (%)	50.5	50.5	50.5	50.5	50.4
Coding DNA Sequences (CDSs)	5430	5608	5555	5504	5587
Protein Coding Region (%)	88.1	86.8	86.9	86.9	86.5
Average ORF length (bp)	896	863	864	868	856
No. rRNA	22	22	22	22	22
No. tRNA	107	109	108	103	100
No. Plasmids	2	2	1	2	1

### Phage Regions in SS17

**Figure 2:**
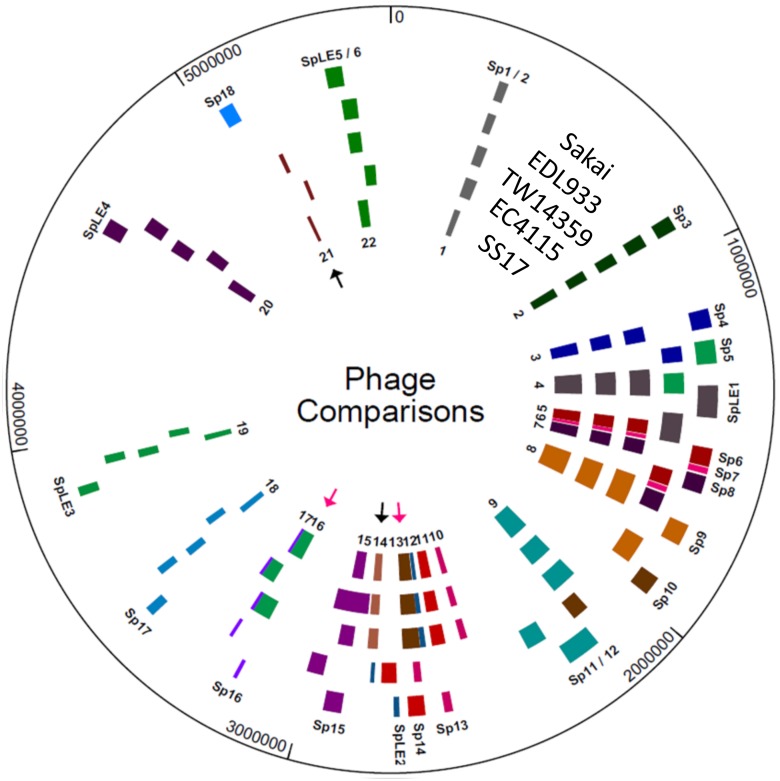
Phage Comparisons between SS17 and reference strains. Location of phage regions were determined in each strain and color coordinated based on insertion sites and similarities. From inner ring: number designation for SS17 phage regions, phage locations: SS17, EC4115, TW14359, EDL933 and Sakai, and S-loop numbers for Sakai phage. Black arrows indicate regions of phage that are present in SS17 and not in EDL933 and Sakai. Pink arrows indicated regions that are translocated in SS17 compared to EDL933 and Sakai.

One of the major mechanisms for genome evolution of O157 is phage-mediated lateral gene transfer, which can include new virulence genes [43]. Analysis of the SS17 genome revealed 22 phage-bearing regions distributed across the entire genome (Fig. 2, Table 2). All of the phage regions encode an integrase and phage assembly proteins. Region 2 contains non-LEE encoded (Nle) secreted effectors, *nleB1*, *nleC*, *nleH1*, and *nleD*, a DNA repair gene, and *lomK*. The non-fimbrial adhesins, *cah* and *iha*, the *ure* and *ter* operons, and *espP* are encoded in the fourth region and the Nle-encoded type III secreted effectors, *espX7* and *espN* are located in the fifth. The non-LEE encoded effectors *nleA*, *nleH2*, and *nleF* along with three tRNAs are located in the eighth region, while a number of non-LEE encoded secreted effectors, including *nleG7*, and a prophage protein, *lomU* are encoded in the ninth region. Region 11 contains 3 tRNAs, *espJ*, and *tccP*, tir-cytoskeleton coupling protein. Region 13 contains the *stx2A* subunit, *stx2Bc* variant and 3 tRNAs, while region 16 contains the *stx2A* and *stx2B* subunits, 3 tRNAs, and *lomW*, an outer membrane protein. Regions 18 and 19 contain the *espM2* and *espW* genes and the *nleB2* and *nleE* genes, respectively. The LEE-encoded genes including *eae*, *tir*, and *ler* are located in region 20, while region 21 contains *lomU* and a phage *eae* gene.

**Table 2:** Genes encoded in the 22 phage regions of SS17.

**Phage Regions**	**Size (bp)**	Encoded genes*
Region 1	24,792	Transposase, invertase, and phage assembly proteins
Region 2	38,592	DNA repair gene, *nleB1*, *nleC*, *nleH1*, *nleD*, *lomK*, and *ykgJ*
Region 3	51,350	Attachment-invasion protein and 2 tRNAs
Region 4	86,713	Colicin immunity protein, *cah*, *iha*, *espP*, *ure operon*, and *ter* operon
Region 5	45,759	*espX7* and *espN*
Region 6	11,206	Phage assembly proteins
Region 7	46,894	Transposase, phage assembly proteins, and *emrE*
Region 8	103,685	*nleA*, *nleH2*, *nleF*, and 3 tRNAs
Region 9	88,439	non-LEE encoded secreted effectors, *nleG7*, *lomU*, and 5 tRNAs
Region 10	22,436	Transposase and phage assembly proteins
Region 11	43,087	Transposase, *espJ*, *tccP*, and 3 tRNAs
Region 12	13,461	Transposase and *yee* operon
Region 13	57,076	*stx2A*, *stx2Bc*, and 3 tRNAs
Region 14	32,418	Phage assembly proteins
Region 15	48,171	Transposase, *emrE*, and 3 tRNAs
Region 16	61,910	Transposase, *stx2A*, *stx2B*, *lomW*, and 3 tRNAs
Region 17	8,551	Phage assembly proteins
Region 18	24,157	Transposase, *espM2*, and *espW*
Region 19	22,139	Transposase, *nleB2*, and *nleE*
Region 20	43,453	Transposase, *eae*, *tir*, *ler, yeeU, and yeeV*
Region 21	12,084	*lomU* and phage *eae*
Region 22	42,885	Phage assembly proteins

* All regions contain at least one integrase

Unlike Sakai and EDL933, SS17 encodes ~54kb at region 13, which is located between *dacD* and *sbcB*. This area includes a copy of the *nleG7* gene, the *stx2c* variant and is similar to the S-loop 10 (Sp10) in Sakai located between genes *ydaO* and *uspF* (Fig. 2, pink arrow). SS17 encodes ~62kb of phage located between *yfdC* and tRNA-Arg at region 16 (Fig. 2, pink arrow). This area has similarity to the Sp5 of Sakai located between *yccJ* and *yccG* and contains the *stx2* A and B subunits. In addition, SS17 contains two phage regions, 14 and 21, that are not in the Sakai or EDL933 genome (Fig. 2, black arrows). Region 14, located between genes *yegQ* and *yegS*, is ~32kb and contains genes coding for an integrase and phage assembly proteins. Region 21 is ~12kb and located between *yjbK* and *dusA*. Analysis shows that this region, even though located proximal to the Sakai’s Mu-like phage containing Sp18, does not have similarity to Sakai’s Sp18 which is absent in SS17. The remaining phage regions are similar in location and size with the exception of region 8, which is ~45kb larger than Sakai’s Sp9 and contains mainly phage assembly and hypothetical proteins, and a Clp protease protein.

### Virulence Genes

**Figure 3:**
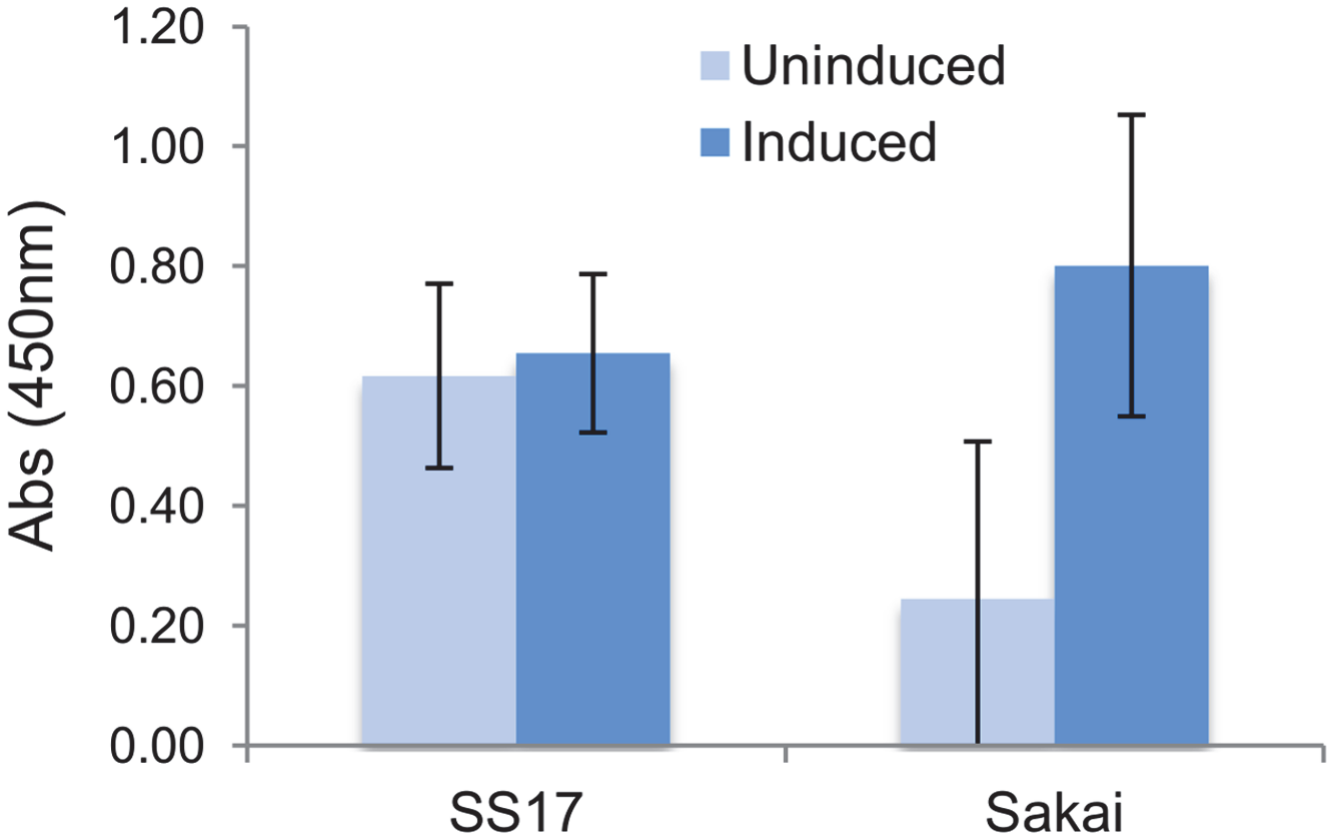
Stx2 induction in SS17 and Sakai strains. The levels of Stx2 produced by SS17 were unaffected by induction and showed higher levels of Stx2 in uninduced cultures when compared to Sakai.

A total of 295 virulence related genes, which have been previously described in other O157 strains were identified in SS17 using an automated clusters of orthologous group (COG) classification together with manual curation (S3 Table) [44]. Many of these genes are involved in multiple functions via various pathogenesis mechanisms employed by O157 during infection, and thus are possible targets for the increased virulence seen in super-shedder isolates. Of these, 276 genes are in the chromosome and 19 are on the pO157 plasmid, and can be broadly divided into three distinct groups based on putative protein function (toxin, adherence, and other virulence-associated). The toxin group consists of 109 genes, including the 47 LEE encoded genes, the 35 non-LEE effectors, and the hemolysin genes, *hlyABCD*, encoded on pO157 [3, 45, 46]. In addition, SS17 encodes for the Shiga toxin (*stx*) genes *stx2a* (*argW* integration) and *stx2c* but lacks the *stx1* gene. Similar to O157 strains EC4115 and TW14359, SS17 has two copies of *stx2A*, one located proximal to the *stx2c* variant, and the other located near *stx2B*. Unlike Sakai, SS17 has the ability to produce Shiga toxin 2 at high levels without induction, suggesting hypervirulence characteristics of this isolate (Fig. 3). Further investigation is required to evaluate the mechanism of this phenotype in relation to super-shedding. In addition to the *stx* genes found on the chromosome, pO157 encodes for the hemolysin genes, *hlyABCD*, which are important for survival of the pathogen in the host [45].

Toxins in SS17, including those that damage host cells or corrupt normal host cellular activities and functions, include the locus of enterocyte effacement (LEE) a well-known feature of O157 strains, and critical for their ability to cause infection in humans [3]. SS17 has 47 LEE encoded genes including the genes encoding secreted effectors, such as *escR* and *escS* in LEE1, and *espF* in LEE4. Additionally, SS17 encodes for 35 non-LEE effectors, including the *nleA-H* genes that also utilize a type III secretion-like system. The few non-LEE proteins that have been studied have been shown to play a role in mediating host responses similar to the LEE genes [46].

**Adherence Genes**. Genes related to adherence and colonization are of particular interest since they may play a role in the unique phenotype exhibited by SS O157. SS17 harbors 107 adherence genes previously identified in other O157:H7 strains. These code for fimbrial and non-fimbrial adhesins, usher proteins, chaperones, regulators, and effectors of adherence. Similar to other O157, the genome of SS17 contains the *fim*, *sfm*, *lpf*, and *csg* fimbrial operons, which have been implicated in the attachment of bacterial cells to many different human cell types [47–51]. These filamentous adhesins are made up of a series of chaperones and usher proteins, and subunits of the fimbrial protein complex. The *fim* operon encodes for the type I fimbriae and the *sfm* operon encodes for a putative chaperone-usher fimbria [48, 49]. The *csg* operon controls biogenesis of curli, the fimbriae known to be important for surfaces attachment and biofilm formation [50].

Non-fimbrial adhesin genes include the LEE5-encoded *eae* (intimin) and *tir* (translocated intimin receptor), and *tccP* (*tir* cytoskeleton coupling protein). These genes and their products are essential in the formation of attaching and effacing (A/E) lesions on epithelial cells. Two other non-fimbrial adhesin genes, *yeeJ* and *yfaL* have been shown to increase the efficiency of adherence to epithelial cells [52]. Also present in SS17 is the iron-regulated adhesin gene, *iha* that has a role in adherence to epithelial cells and serves as an enterochelin transporter [53]. This protein has the potential to aid in the adherence and colonization of the bacteria to host cells and concomitantly in iron acquisition. Numerous usher proteins, chaperones, and regulators associated with both fimbrial and non-fimbrial adhesins are found in the SS17 genome. Genes include the fimbrial usher, *htrE*, the pilin chaperone, *sfmC*, the type I fimbriae, *fimC*, and the global transcriptional regulators *crl* and *hha* [54, 55]. Crl controls the curli operon, which has a second regulator, *csgD* and a transcriptional dual regulator, *fur* [55]. Hha controls flagellar and curli gene expression, but is also an important repressor of the hemolysin genes [54]. In addition to adhesins and their regulators, effectors of adherence also play an important role. For instance, the pO157 contains the *etp* operon, which codes for the type II secretion pathway and has a number of adherence related effectors [56].

The remaining 79 genes are involved in regulating virulence as well as survival in the host through evasion of host defenses and biofilm formation. Genes include the *ter* and *sap* operons, which are dedicated to protecting the bacteria against phage, colicins and tellurite, and human-derived antimicrobial peptides, and the *mot* and *fli* operons, which control the expression and function of the flagella and have implications in both motility and adherence [57]. In addition, SS17 encodes the quorum sensing systems, *sdiA*, *luxS*, *qseE/F* with auto-inducers 1, 2, and 3, respectively. Quorum sensing has been described in reference strains as being tightly linked to the development of biofilms [58]. SS17 also harbors the *wca* and *pga* operons involved in biofilm and capsule biogenesis, and the *chu* operon involved in iron sequestering.

### Evolutionary and Phylogenetic Analysis of SS17

SS17 genotyping was performed to determine if the bovine super-shedder isolate is characteristic of bovine isolates or human clinical isolates. We first looked at the *tir* SNP, which is either an A or T at base 255. Studies in the United States and the Netherlands have shown that the *tir* A and T alleles are present in equal proportions in bovine isolates, with the T allele in the majority of food isolates (~62%) and overrepresented in human isolates (>93%) [59, 60]. Our analysis confirms that SS17 contains the T allele, and is more closely related to clinical O157 isolates than bovine isolates based on this SNP, which has a higher tendency to cause disease in humans.

Phylogenetic grouping has been used to investigate the evolution of pathogenic *E. coli* and allows the separation of strains into four groups (A, B1, B2, and D) based on the presence of *chuA*, *yjaA*, and a DNA fragment, TspE4.C4 [61]. SS17 belongs to the D group as the isolate contains the *chuA* gene, but not *yjaA* or TspE4.C4. Studies have shown that commensal strains belong to the A and B1 groups and virulent strains belong to the B2 and D groups [61, 62]. In addition, it was reported that the majority of isolates in the D group came from humans whereas cattle isolates were most prevalent in the B1 group [63].

Further analysis of the genome sequence placed SS17 in clade 8 based on the presence of a SNP (3468C) in the *rhsA* gene [60, 64]. When compared to clade 2, clade 8 strains have been shown to have 2-fold greater adherence to bovine mammary epithelial cells (MAC-T) and increased expression of virulence genes, including the LEE-encoded genes, *espAB*, *tir*, and *eae*, as well as *stx2*, and the plasmid encoded *hlyA*, *toxB*, and *tagA* [65]. Studies have shown that clade 8 O157 strains can be categorized as lineage I/II [66, 67]. Lineage classification can also be used to study the evolution of strains and is determined by the lineage-specific polymorphism assay (LSPA), which divides strains into lineage I, lineage II, or lineage I/II based on six polymorphic loci [39, 68]. The analysis classified SS17 as lineage I/II based on its LSPA profile (211111). An additional phylogenetic typing method that has been shown to be closely aligned with lineage classification, is the distribution of the insertion sequence 629 (IS*629*) [69]. Along with stx genotyping, the distribution of IS*629* in SS17 shows a segregation with the lineage I/II strains (S4 Table), supporting the classification of SS17 as lineage I/II. Lineage I/II contain strains linked to spinach-associated outbreaks [27].

Shiga toxin-encoding bacteriophage insertion sites (SBI) is another typing method that has been used to cluster STEC isolates based on the presence of the intact or occupied insertion sites, *yehV* and *wrbA* [70]. Genotypes designated as 1, 2, and 3 account for 95% of the human clinical isolates and only 51% of bovine isolates. In the genome of SS17, the *yehV* insertion site is occupied and the *wrbA* insertion site is intact, which along with encoding stx2, places SS17 into genotype 1.

Overall, SS17 has more characteristics common among human isolates of O157 than bovine isolates and has a number of markers that suggest increased virulence.

### Whole Genome Comparison

**Figure 4:**
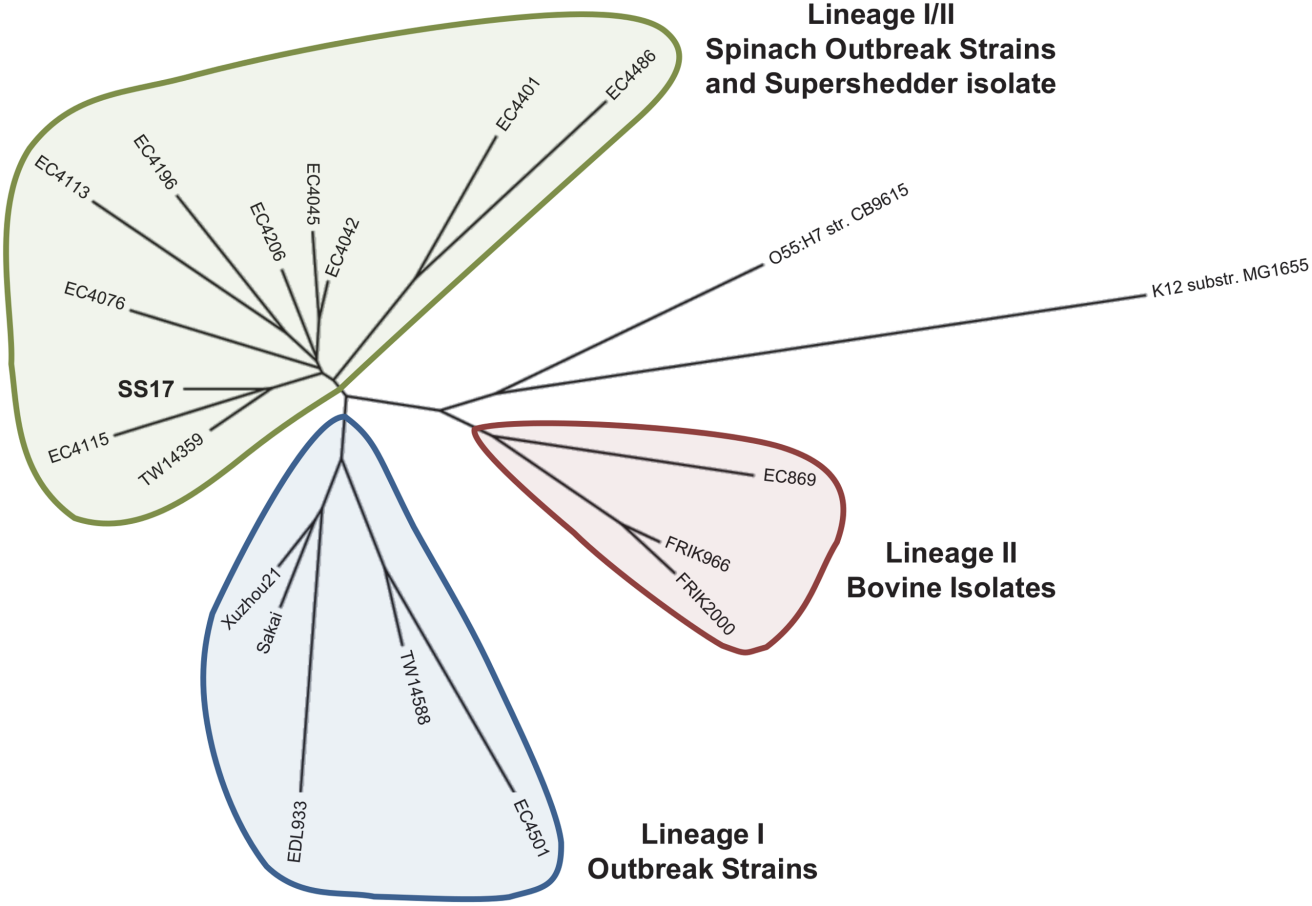
Cladogram of SS17 and O157 outbreak strains. Whole genome analysis shows SS17 clusters with the lineage I/II spinach outbreak isolates (EC4115 and TW14359). This indicates a closer relationship with these strains than with the lineage I outbreak isolates (Sakai and EDL933) and the bovine lineage II isolates.

One of the important aspects of this study was to determine genomic level factors that have the potential to contribute to the super-shedder phenotype, specifically genetic loci that can be used to distinguish and define super-shedder isolates. However, it is very difficult to distinguish a non-super-shedder from a super-shedder isolate since fecal shedding levels are temporal and can vary from animal to animal [13]. For instance, the level of shedding usually increases during the warmer months, whereas the colder months see a decrease in shedding. Moreover, a low shedding isolate may be obtained during the same season and even at the same time as a high shedder and still not be considered a non-super-shedder as the peak shedding may not have been reached at the time of sampling. With this in mind, we used the previously sequenced O157 genomes as references that were considered to be distinct from SS17 according to the whole genome clustering (Fig. 4) [27–30]. We performed whole genome comparisons and identified polymorphism unique to SS17, concentrating on those polymorphisms that are in virulence related genes.

We examined the relatedness of the genome of SS17 and O157 strains with sequence information available, including those associated with foodborne outbreaks and those isolated from bovine sources. This whole genome analysis revealed a close clustering of SS17 with the lineage I/II strains including the spinach-associated outbreaks strains TW14359 and EC4115, and segregation from the lineage I O157 strains, such as Sakai and EDL933, and the lineage II bovine isolates (Fig. 4). This is consistent with the LSPA analysis, which classifies SS17 into lineage I/II and with the close homology of SS17 with the clonal isolates, TW14359 and EC4115, seen in the whole genome comparisons.

**Figure 5:**
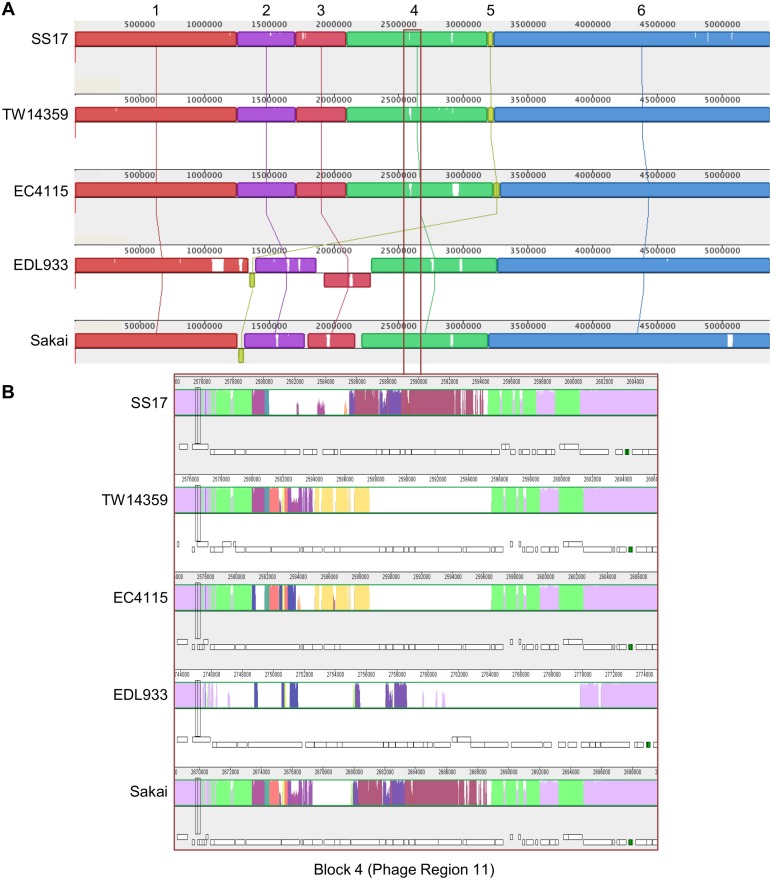
Mauve alignments of SS17 with the four reference genomes. (A) Alignment of SS17 with the four reference strains (TW14359, EC4115, EDL933, and Sakai) reveal six blocks of homology each of varying length ranging from ~50kb to ~2280kb. Each block is a different color with lines connecting corresponding homologous blocks, with the white regions indicating non-homology. (B) Part of block 4 corresponding to phage region 11 is enlarged indicating the differences in the varying colors between SS17 and the reference strains which can also be seen in the annotations (white bars underneath).

To determine differences in SS17 as compared to the reference O157 strains across the whole genome, we used Mauve to assign the genomes into blocks of homology [37, 38] (Fig. 5). When SS17 is compared to the reference strains (TW14359, EC4115, EDL933, and Sakai), there are 5,121,820 bp conserved among all isolates (~93%). In addition, there is approximately 42 kb of non-homologous regions in SS17, ranging from 20 bp to 6,296 bp with an average length of 822 bp. The majority of the differences seen in the alignment originate from phage coding regions.

Mauve alignments of SS17 with the four reference genomes revealed six blocks of homology with lengths ranging from ~50 kb to ~2280 kb. The first block of ~1250 kb contains four areas that are non-homologous in phage regions and includes part of the phage region 3 (Fig. 2). In the second homology block of ~460 kb, five areas show non-homologous phage regions and include phage region 5, 6, 7, and part of 8 (Fig. 2). The third ~400 kb block, which is inverted in EDL933, contains three regions that display non-homologous regions of phage among all of the reference strains and includes the remaining phage region 8. The fourth ~1090 kb block of homology contains nine regions that are non-homologous when SS17 is compared to the reference strains (Fig. 5). Phage regions 11–15 are contained within this block of homology (Fig. 2). Phage region 13 is one of the two regions that are translocated in SS17 when compared to Sakai and EDL933 (Fig. 2, pink arrow), while phage 14 is one of the two not found in the genome of Sakai or EDL933 (Fig. 2, black arrow). The smallest block (five) contains the *stx2* gene and is similar to TW14359 and EC4115 and inverted in EDL933 and Sakai. Part of phage region with the ~400 kb inversion in EDL933 spaning the replication terminus [30]. The block also contains two transposases that are not found in EDL933 and Sakai. Phage 16 is located at the end of this block and contains the *stx2* A and B subunits. This phage region is the second region that is translocated in SS17 with similarity to the Sp5 of Sakai (Fig. 2, pink arrow). The sixth block at ~2280 kb is the largest and contains phage regions 16–22. Phage region 21 is the second phage region not found in Sakai or EDL933 but has similarity to TW14359 and EC4115 (Fig. 2, black arrow).

We also compared the plasmids present in SS17. The pO157 in SS17 is similar to the pO157 found in TW14359 and EC4115, and differs from the plasmid in Sakai in three regions. Two regions contain additional genes not found in Sakai (2 transposase genes, and a transposase and 2 hypothetical proteins), while the third region, contains a reverse transcriptase gene in Sakai that is absent in SS17. pSS17 plasmid is similar to EC4115 with only minor differences.

### Single Nucleotide Polymorphisms

**Figure 6:**
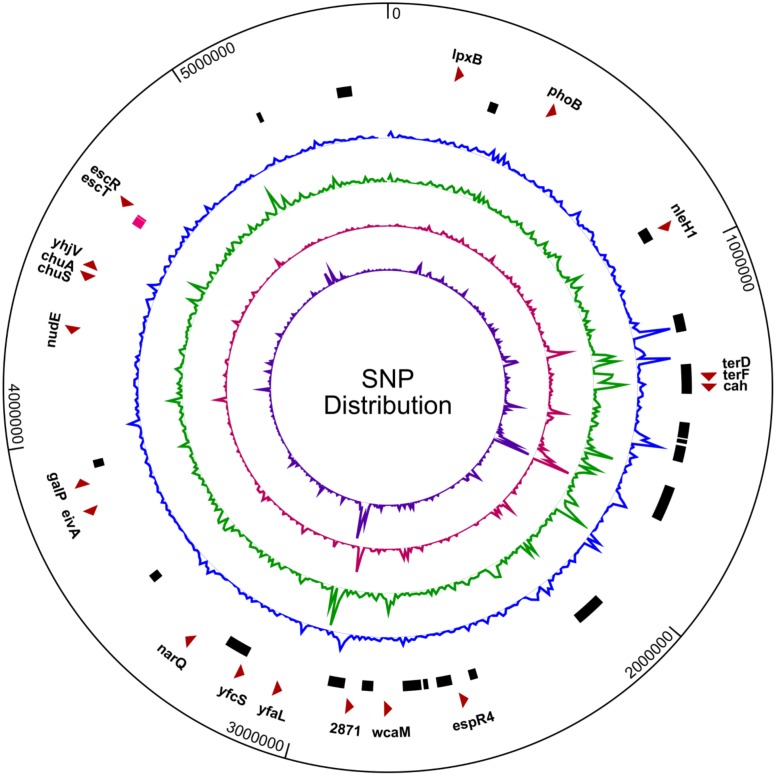
SNP distribution in the SS17 genome. Distribution of single nucleotide polymorphisms (SNPs) in the SS17 genome shows a high density of SNPs clustered in phage regions. From outer ring: select genes with nsSNPs (red arrows), SS17 phage regions (black bars) with LEE operon (pink bar), SNP distribution when compared to Sakai (blue), EDL933 (green), TW14359 (red), and EC4115 (purple).

The analysis revealed 310 SNPs in SS17 as compared with EC4115, 833 SNPs as compared with TW14359, 3860 as compared with Sakai, and 4847 as compared with EDL933 (S1 Fig., S5 Table). The majority of these SNPs are located in phage regions (Fig. 6). There are 147 genes that have 262 non-synonymous SNPs (nsSNPs) unique to SS17 and not found in the reference strains. A few examples of genes with nsSNPs include *narQ*, *galP*, *yhjV*, and *nudE* (Fig. 6). The *narQ* gene along with *narP* belongs to the two-component sensor-histidine kinase system and has one nsSNP of T452I. The *galP* gene, which belongs to the galactose transport family, has one nsSNP with two nucleotide substitutions at L433H. The GalP protein is a low-affinity H+ symporter of D-galactose (and glucose) used by *E. coli* in metabolic and catabolic pathways including glycolysis [71, 72]. Previous studies have shown that the glucose utilization rate in *E. coli* correlates directly with the expression levels of *galP* and strains with modulated GalP showed an increased glucose utilization rate with a positively correlated gene expression strength [73, 74]. The *yhjV* gene, an uncharacterized member of the STP family of transporters, has one sSNP and three nsSNPs at L103F, I109S, and N111I. The *nudE* gene belongs to the nudix hydrolase family of genes and has one nsSNP with one nucleotide substitutions at P146H.

There are 27, 13, and 8 nsSNPs in genes related to adherence, toxin production, and other virulence functions, respectively, when comparing SS17 with reference genomes. In each of these categories, there are 20, 13, and 8 genes, respectively. The majority of the nsSNPs (27) are located in genes responsible for adherence, specifically non-fimbrial adhesins and include *cah*, *yfaL*, and *toxB* (Fig. 6, S6 Table). Of particular interest is the *cah* gene, which encodes the calcium-binding antigen 43 homologue and is an autotransporter that can function in adherence, autoaggregation, and biofilm formation [75]. There is a nonsense mutation that causes the terminal end of the gene to be truncated 170 amino acids shorter than reference O157:H7 strains. This is in the β-barrel domain of the protein, which is inserted into the membrane to mediate the export of the processed adhesin portion of the protein [75]. Deleting the *cah* gene in EDL933 has been shown to reduce biofilm formation and the autoaggregative phenotype but not eliminate the ability of the bacteria to adhere to human Caco-2 and HeLa cells, or to alfalfa sprouts [75, 76]. The *yfaL* gene encodes an autotransporter type V AIDA-like adhesin and functions in the formation of biofilms, cell-to-cell aggregation. When over-expressed, YfaL can contribute to adherence to abiotic surfaces, including plastic [77]. There are three nsSNPs in the SS17 *yfaL* gene when compared to EDL933 and Sakai, with one in the N-terminal signal region and two in the passenger region [52]. The signal peptide allows for the correct localization of the protein while the passenger region is cleaved and folded to create extracellular effectors to provide the functional and virulence properties of the protein [78]. There is one adherence-related gene, *toxB*, located on pO157 which contains two nsSNPs and three synonymous SNPs. The gene functions in adherence to epithelial cells and is a homolog to the *efa1* gene found in non-O157 strains. It has been demonstrated that deletion of the *toxB* gene causes a reduction in adherence to epithelial cells and reduced expression and secretion of LEE-encoded proteins, specifically those in the LEE4 operon [79].

There are also 13 nsSNPs in toxin genes including the LEE-encoded type III secretion factors (*escT* & *escR*), the non-LEE mediated host cell effector (*espR4* & *nleH1*), the lipid A biosynthesis gene (*lpxB*), and plasmid-borne hemolysin genes (*hlyA* & *hlyC*). The virulence-associated category has 8 nsSNPs and includes genes involved in nutrient acquisition (*chuA* & *chuS*), capsule biogenesis (*wcaM*), resistance (*terD* & *terF*), and signaling (*phoB*) (Fig. 6).

When compared to all four O157 reference strains, there are three adherence genes (*cah*, *yfcS*, & SS17_2871), two toxin-related genes (*eivA* & *lpxB*), and two virulence-associated genes (*chuA* & *chuS*) with nsSNPs (S6 Table). The *yfcS* gene is a predicted periplasmic pilus chaperone and SS17_2871 is a fimbrial-like adhesin, and both contain one nsSNP.

### Other Polymorphisms

In addition to analyzing genes for polymorphisms and protein changes, we screened upstream (250bp) and downstream (100bp) sequences to determine any possible change in promoter or termination regions that could affect protein expression and contribute to the super-shedder phenotype. Our analysis revealed 108 genes with different upstream sequences compared to the four O157 reference strains. These genes included the adherence related *eaeH*, *csgD*, *wzzB*, and *yraH*, the LEE-encoded type III effector, *espG*, and the auto-inducer, *luxS*, besides the membrane proteins, *ytfF* and *imp*, a putative membrane permeability protein, and an outer membrane receptor, *fepA*. Downstream, terminator sequence analysis revealed 95 genes with polymorphisms compared to all four of the O157 reference strains. These genes included the outer membrane proteins, *ompF*, *yhfL* and *lomW*, the iron-siderophore receptor precursor, *fhuE*, the regulatory protein, *abgR*, and *yeeV*, which is the toxin component of the YeeV-YeeU toxin-antitoxin system.

There are 23 genes with one or more amino acids that have been deleted or inserted in SS17 and include mainly hypothetical and phage-related proteins. The *mdtO* gene, part of the multidrug efflux family of proteins that are involved in resistance to puromycin, acriflavine, and tetraphenylarsonium chloride, is one of the genes with a deletion of 3 amino acids in the middle of the protein sequence. In addition, the *appB* gene, a cytochrome d ubiquinol oxidase, has eight nsSNPs and one inserted amino acid. Out of the 5,430 CDS in SS17, 3 genes (*hflK*, *hyfA*, and *ybhF*) have mutations that resulted in frameshifts. The gene *hflK* is a protease regulator, *hyfA*, a hydrogenase part of a ten-gene cluster that interacts with formate dehydrogenase, and *ybhF* is an ABC transporter of the multidrug efflux pump [80].

### Adherence of SS17 to Bovine RAJ Cells

**Figure 7:**
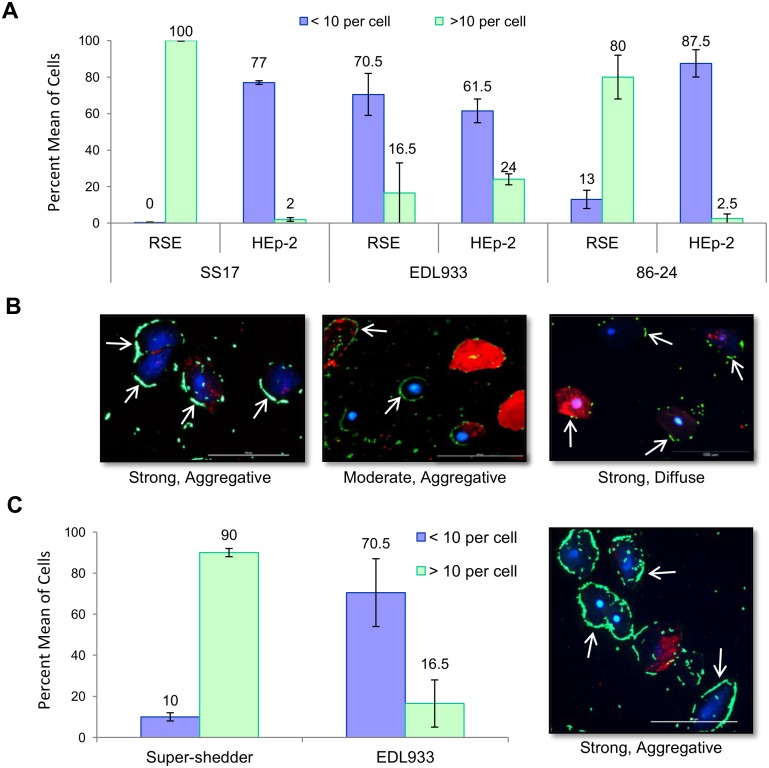
Adherence patterns of O157 strains SS17, EDL933, and 86–24-Sm^R^ to RSE cells. (A) Quantification of SS17, EDL933, and 86–24-SmR adherence to RSE and HEp-2 cells in the presences of D+Mannose. SS17 displays a strong adherence to RSE cells and only moderate adherence to HEp-2 cells. (B) Immunofluorescence stained slides reveled that both SS17 and EDL933 displayed an aggregative adherence pattern on RSE cells while 86–24 displayed a diffuse pattern. All strains demonstrated a diffuse adherence pattern on HEp-2 cells (not shown). (C) Average of adherence levels for nine super-shedder isolates, which all displayed strong, aggregative adherence to RSE cells, that can be seen in the representative immunofluorescence stained slide. Arrows point to adherent bacteria (green). Slides are shown at 40x magnification with RSE cytokeratin (red) and nuclei (blue).

Since the RAJ is the primary colonization site of O157, we examined the ability of super-shedder isolates to adhere to RSE cells isolated from the RAJ region. SS17 interacted with bovine RSE cells in a distinct adherence pattern when compared to the O157 strains, EDL933 and 86–24-Sm^R^, in the presence of D+Mannose (Fig. 7 A and B, S7 Table). The results show that 100% of the cells exposed to SS17 have more than 10 adhering bacteria cells per RSE cell, which is significantly (p < 0.01) greater than EDL933 (16.5%) and 86–24-Sm^R^ (80%). In addition, immunofluorescent microscopy shows SS17 forming aggregates on the RSE cell surface (Fig. 7B). Interestingly, this strong, aggregative adherence pattern of SS17 on RSE cells was different from the moderate, diffuse adherence pattern observed for the same isolate on human HEp-2 cells (Fig. 7A). In addition to SS17, eight other super-shedder isolates demonstrated this unique adherence pattern (Fig. 7C, S7 Table). This suggests a unique phenotype in super-shedder isolates that allows greater adherence to the bovine rectal epithelial cells.

### Role of LEE in SS17 Adherence to Bovine RSE Cells

Experimental evidence has shown the LEE, in particular the LEE4 operon encoding for EspA, EspB, EspD, Tir, and other secreted factors, to be essential for the adherence and colonization of O157 to FAE cells [81]. LEE4 deletion mutants demonstrated decreased adherence to FAE cells *in vitro* and decreased fecal shedding in orally inoculated calves [81]. In order to evaluate the involvement of LEE proteins in the adherence of O157 to RSE cells, Kudva *et al*, used antisera against intimin, Tir, EspA and EspB, and an intimin knockout mutant, and determined that the lack of these proteins did not block the adherence of O157 to RSE cells, indicating a LEE-independent mechanism of adherence [40]. To elucidate to possible mechanisms used by O157 to adhere to RSE cells, the proteome of O157 was defined under the adherence assay conditions [40]. An evaluation of the 684 expressed proteins identified in the proteome revealed the presence of 36 potential adhesins of which three potential adhesins (ChuA, TerE, and a putative outer membrane porin protein) were unique to O157 [40]. It remains unknown what role the expressed adhesins play in the adherence of O157 to RSE cells and if super-shedder isolates use a similar or a different mechanism of adherence.

**Figure 8:**
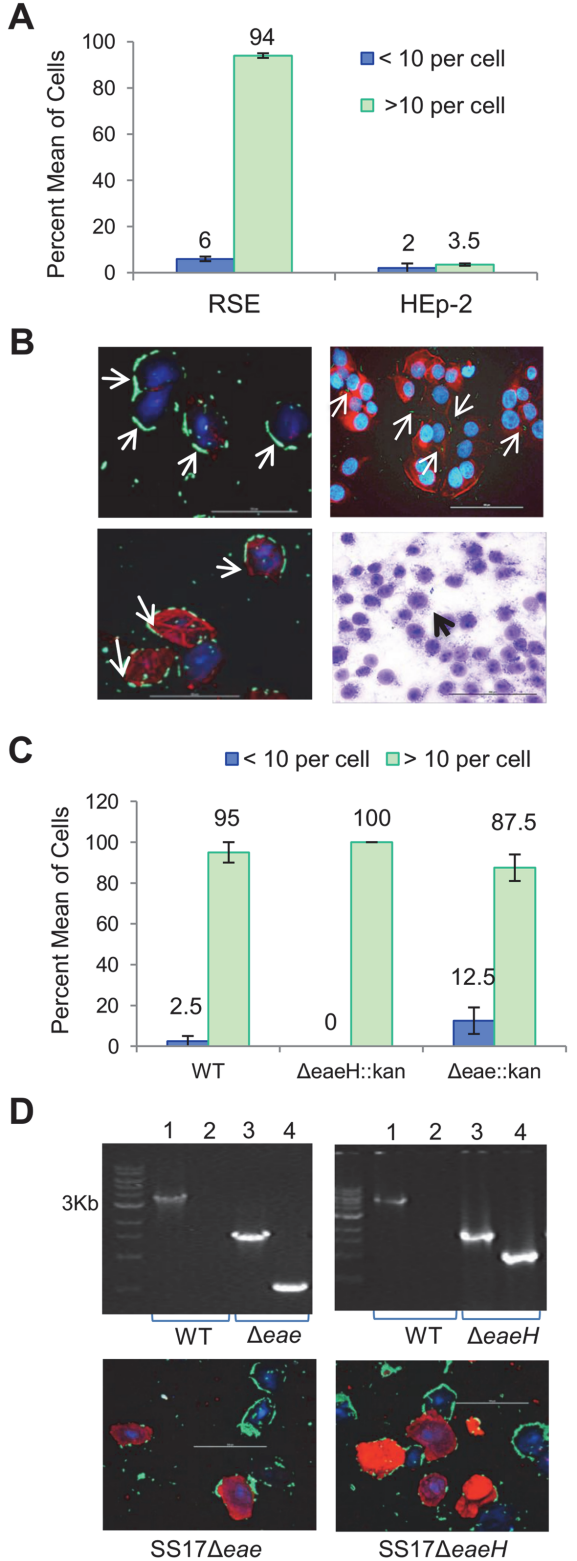
Adherence pattern of SS17 on RSE and HEp-2 cells in the presence of antisera. Pooled antisera against LEE-encoded proteins (EspA, EspB, Tir, and Intimin) were used to elucidate to mechanisms of adherence. (A) Adherence of SS17 on bovine RSE cells revealed a LEE-independent mechanism while decreased adherence of SS17 on human HEp-2 cells showed a LEE-dependent mechanism of attachment. (B) Top panel, immunofluorescence (IF) stained images show adherence of SS17 to RSE cells and HEp-2 cells in the absence of antisera. Bottom panel, IF slide shows SS17 attachment to RSE cells, and the toluidine blue (TB) stained slide lack of adherence of SS17 to HEp-2 cells, in the presence of antisera. (C) Quantification of SS17 wild type, SS17Δeae, and SS17ΔeaeH to RSE cells, displaying no change in the adherence characteristics of mutants in comparison to wild type SS17. (D) Top panel. SS17Δeae and SS17ΔeaeH were confirmed by PCR. Lane 1 represent the gene of interest in wild type SS17, lane 2 the absence of the kanamycin gene in wild type SS17, lane 3 the gene of interest in the mutants and lane 4 the insertion of the kanamycin cassette in the mutants. (D) Bottom panel. Immunofluorescence stained slides indicate no change in adherence for SS17Δeae and SS17ΔeaeH mutants on RSE cells. All slides are at 40x magnification, and have arrows pointing to SS17 bacteria (green); with RSE cytokeratins and HEp2 actin filaments in red, and nuclei of both cells in blue.

To understand possible mechanisms involved in the increased adherence of SS17 to RSE cells, pooled antisera against the LEE-encoded proteins, intimin, Tir, EspA, and EspB, was used to inhibit this adherence pathway. The results showed that the pooled antisera did not block the adherence of SS17 to RSE cells but did block the adherence of SS17 to HEp-2 cells causing SS17 to be ‘non-adherent’ (Fig. 8A–B, S7 Table). A negligible (6%) decrease in adherence to RSE cells was observed in the presence of the antisera. This indicates that SS17 uses a mechanism independent of these LEE proteins.

To further assess the role of LEE in the hyper-adherence phenotype exhibited by SS17 to the RSE cells, and the molecular mechanisms involved, we constructed SS17Δ*eae* and SS17Δ*eaeH* mutants (Fig. 8D). SS17 encodes for *eae* and its homologue *eaeH*, both of which are known to be involved in LEE-dependent adherence of O157 to various host cells [82, 83]. The adherence characteristics of these mutants were assayed using the RSE model and both SS17Δ*eae* and SS17Δ*eaeH* mutants did not show any change in their ability to adhere to RSE cells as compared with the wild type (Fig. 8C–D). However, since these genes are homologues and can have redundant functions, further studies using double mutants will be needed to confirm their exact role in mediating adherence to bovine RSE cells.

Finally, the role of type 1 fimbriae in the adherence of SS17 to RSE cells was investigated based on the observation that type 1 fimbriae adherence is inhibited in the presence of D+Mannose [20]. Our results show that there is no difference in adherence levels of SS17 to RSE or to HEp-2 cells in the presence or absence of D+Mannose, indicating that type 1 fimbriae are not being utilized for adherence to these cell types (Fig. 7). The results from these mechanistic studies are consistent with the prior speculation that unique mechanisms or factors distinct from LEE-encoded proteins may be responsible for the adherence of SS17 and other O157 strains to bovine RSE cells [40].

## Conclusions

*E. coli* O157:H7 remains a major foodborne illness concern even with the implementation of numerous antimicrobial interventions in beef processing plants and the recent development of novel pre-harvest control methods including cattle vaccination and bacteriophage application on to the animal hide [11, 16, 84, 85].

With the major animal reservoir being asymptomatic cattle, the greatest challenge is to effectively control the pathogen in large populations of cattle housed in close proximity in the absence of symptoms of infection. Reducing the pathogenic bacteria load in cattle is the most direct approach to reduce environmental contamination, human exposure, and ultimately foodborne illness and death [86]. While a number of approaches including vaccination, the use of probiotics, and the application of bacteriophage have been tested with the goal of reducing O157 in cattle, these have unfortunately met with only limited success [11, 16, 84, 85]. One possible explanation is that these methods do not take into account the small population of animals that are colonized by super-shedder isolates of O157 which are the largest contributor of *E. coli* to the environment and account for the majority of transmission events [11].

An optimal control method would be one that is cost effective to be applied to a large population of cattle and yet sufficiently specific enough to target super-shedder isolates. In order to control super-shedder isolates in cattle, treatment methods will need to focus on preventing the bacteria from adhering and colonizing the recto-anal junction (RAJ), as this is a super-shedder related microbial factor that is known to contribute to high fecal shedding [22].

Although various environmental and cattle host factors have been studied and are likely to contribute to super-shedding of O157 by cattle, little is known about the microbial factors and molecular mechanisms that contribute to this phenotype [13]. With this in mind, we set out to analyze the genome of a super-shedder isolate and identify specific genomic factors and loci that could be further studied to determine their role in the super-shedder phenotype. Our analysis has revealed approximately 60 targets that deserve further examination to determine the specific effect, if any, that may contribute to the super-shedder phenotype. Targets include those that contain nsSNPs and other polymorphisms in virulence genes, specifically those involved in adherence. Since SS17 has a genome arrangement and composition that is similar to other isolates of O157, large rearrangements and insertions or deletions are not expected to be the cause of the super-shedder phenotype. More likely, the numerous nsSNPs in virulence and adherence genes may play a role in the phenotype. Given the distinct strong, aggregative adherence pattern observed with SS17 and other super-shedder isolates on RSE cells, it would be relevant to dissect mechanisms enabling this type of unique adherence. LEE-encoded proteins, critical to O157 interactions with FAE cells and HEp-2 cells [10, 81], were found not to contribute towards the adherence of SS17 to RSE cells. In addition, a role for type I fimbriae was ruled out by the lack of adherence inhibition observed in the presence of D+Mannose. This was not unexpected as other O157 strains, including EDL933 and 86–24, displayed adherence patterns that were not affected by LEE antisera or D+Mannose [40]. Furthermore, *eae* and it homologue *eaeH* did not play a role in the increased adherence of SS17 to RSE cells as there was no change in adherence levels when SS17 mutants of these genes were tested. Understanding the molecular mechanisms of super-shedder adherence to RSE cells is critical for the development of control strategies that target bacterial adherence and can be used to eliminate O157 from the RAJ, and thus prevent the transmission within cattle herd, and ultimately the spread to humans.

The study presented here and future studies will enable the identification of the mechanism(s) involved in the adherence and colonization of the bovine RAJ. These studies will also aid in the overall goal to determine the molecular mechanism or factors that could possibly contribute to super-shedding at the microbe level. Knowing the cause of super-shedding at the microbe level will enable the design of novel/alternative methods that can target super-shedder isolates of O157 to reduce this pathogenic bacteria, prevent the transmission within cattle herds, and ultimately prevent the spread to humans.

## Supporting Information

S1 Fig SNP pattern of reference O157 genomes compared to SS17. Single nucleotide polymorphisms (SNPs) in SS17 compared to Sakai (blue), EDL933 (green), TW14359 (red), and EC4115 (purple).
(TIFF)

S1 Program Perl Script used to identify loci that were mutated in SS17. (ZIP)

S1 Table MLST profile of SS17 and reference O157 strains. (XLSX)

S2 Table Primers used to generate knockout mutants. (DOC)

S3 Table Virulence genes encoded in SS17 with COG analysis. (XLSX)

S4 Table SS17 IS*629* distribution profile. Table adapted from Stanton, *et al*. 2014.
(XLSX)

S5 Table SS17 SNP panel. (XLSX)

S6 Table SS17 virulence genes with nsSNPs. (DOCX)

S7 Table Quantification of SS17 RSE adherence assay. (XLSX)
