# Supplementary figures and images for: Comparative Analysis of Super-Shedder Strains of Escherichia coli O157:H7 Reveals Distinctive Genomic Features and a Strongly Aggregative Adherent Phenotype on Bovine Rectoanal Junction Squamous Epithelial Cells
Source: PLoS One. 2015 Feb 9;10(2):e0116743. doi: 10.1371/journal.pone.0116743 (PMC4321836; doi:10.1371/journal.pone.0116743)

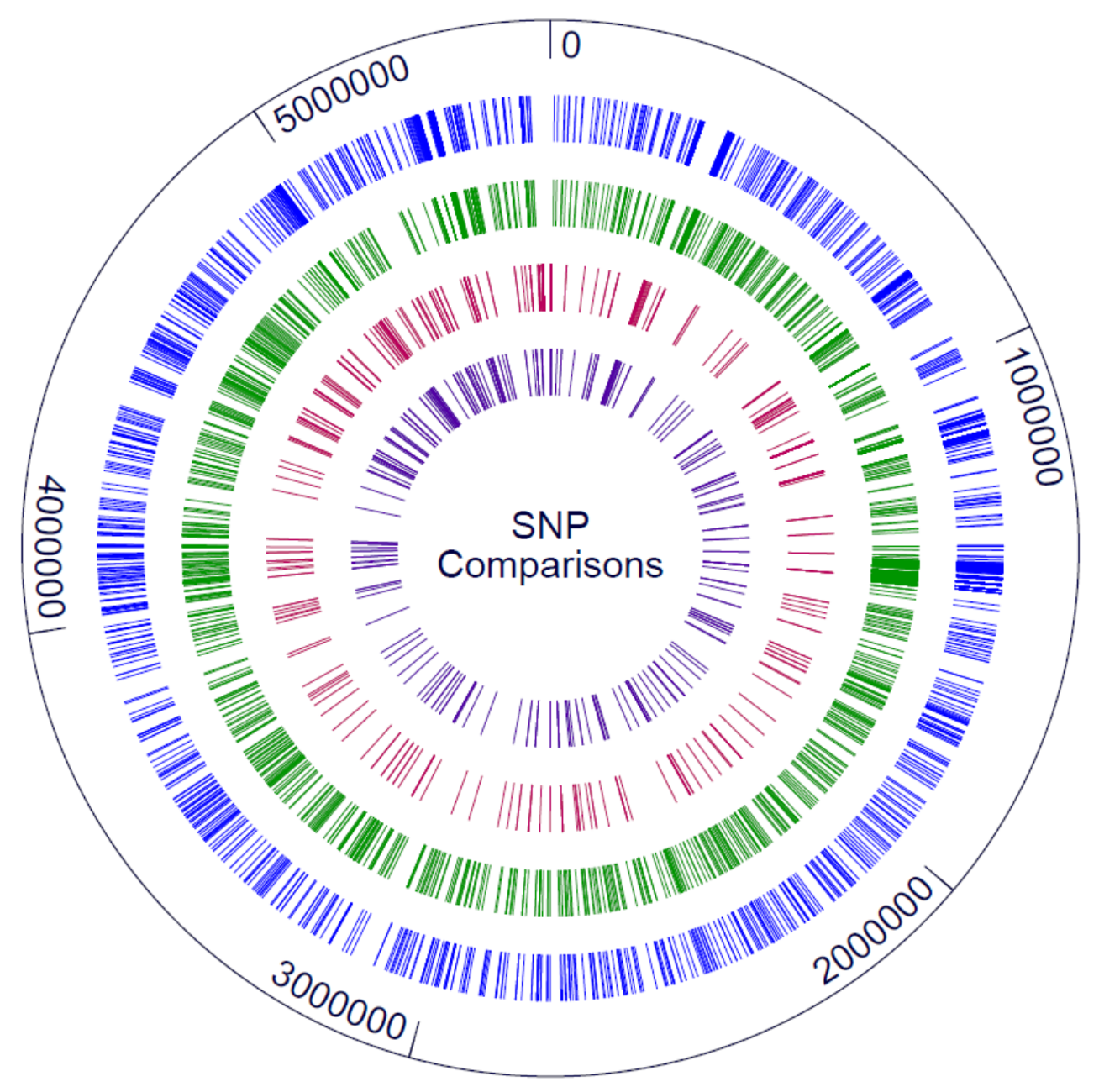

Supplement: S1 Fig — Single nucleotide polymorphisms (SNPs) in SS17 compared to Sakai (blue), EDL933 (green), TW14359 (red), and EC4115 (purple). (TIFF) [file pone.0116743.s001.tiff]
