# Supplementary material for: Comparative Analysis of Super-Shedder Strains of Escherichia coli O157:H7 Reveals Distinctive Genomic Features and a Strongly Aggregative Adherent Phenotype on Bovine Rectoanal Junction Squamous Epithelial Cells
Source: PLoS One. 2015 Feb 9;10(2):e0116743. doi: 10.1371/journal.pone.0116743 (PMC4321836; doi:10.1371/journal.pone.0116743)
Supplement: S2 Table — (DOC) [file pone.0116743.s004.doc]

Table S2: Primers used to generate knockout mutants.

| **Primer Name** | **Gene** | **Primer Direction** | **Sequence*** |
| --- | --- | --- | --- |
| Eae-Kan_F | *eae* | Forward | ttggtatcagcgtggttggatcaacctacatgagaaaacgtgaatgtgtc  TCCCGTCAAGTCAGCGTAAT |
| Eae-Kan_R | *eae* | Reverse | atccgatctattaatataatttatttctcattctaactcattgtggtgga  CAACAAAGCCACGTTGTGTC |
| EaeH-Kan_F | *eaeH* | Forward | tatttgtgtctgcctatgttcgttaattcgttcatcaggaaattatctca  CAACAAAGCCACGTTGTGTC |
| EaeH-Kan_R | *eaeH* | Reverse | tcaacgccattattgtttattagaatgttacttccatattcttaatatta  TCCCGTCAAGTCAGCGTAAT |
| Eae-A | *eae* | Forward | CTCAGAATCAGCGGTACGATG |
| Eae-B | *eae* | Reverse | CGTTACGTTTCCCTCTCGATG |
| EaeH-A | *eaeH* | Forward | GCGATGGGTAGTGCAAGTTC |
| EaeH-B | *eaeH* | Reverse | CTATGAAGGTGAGTGGGAGC |
| KanC | *KanR* | Forward | GTATTTCGTCTCGCTCAGGC |
| KanD | *KanR* | Reverse | GATGTTGGACGAGTCGGAAT |
| KanC2 | *KanR* | Forward | GCCTGAGCGAGACGAAATAC |
| KanD2 | *KanR* | Reverse | GATGTTGGACGAGTCGGAAT |

* The 50bp gene homology is indicated in lower case
