# Supplementary material for: Comparative Analysis of Super-Shedder Strains of Escherichia coli O157:H7 Reveals Distinctive Genomic Features and a Strongly Aggregative Adherent Phenotype on Bovine Rectoanal Junction Squamous Epithelial Cells
Source: PLoS One. 2015 Feb 9;10(2):e0116743. doi: 10.1371/journal.pone.0116743 (PMC4321836; doi:10.1371/journal.pone.0116743)
Supplement: S6 Table — (DOCX) [file pone.0116743.s008.docx]

Table S6: Virulence genes in SS17 with nsSNPs

| **Category & Location** | **Gene** | **SNP** | **Reference*** | **Product** | **Role in Virulence** |
| --- | --- | --- | --- | --- | --- |
| Toxin |  |  |  |  |  |
| Chromosome | *eivA* | P33S | 5 | NLE T3SS apparatus protein | LEE-encoded effector secretion |
|  | *eivF* | H163Q | 3 & 4 | Putative regulatory protein  for T3SS | Functional regulation |
|  | *escE* | H46N | 4 | LEE T3SS factor | LEE-encoded effector secretion |
|  | *escR* | G29V | 4 | LEE T3SS factor | LEE-encoded effector secretion |
|  | *escT* | F68L | 1 | LEE T3SS factor | LEE-encoded effector secretion |
|  | *espR1* | S57N | 2 | Predicted NLE T3SS effector | NLE mediated host cell effects |
|  | *espR4* | S93A | 3 & 4 | Predicted NLE T3SS effector | NLE mediated host cell effects |
|  | *espX6* | F220L | 3 & 4 | Predicted NLE T3SS effector | LEE mediated host cell effects |
|  | *lpxB* | A181R | 5 | Tetraacyldisaccharide-1-P synthase | Endotoxin Biosynthesis |
|  | *nleH1* | V245I | 3 & 4 | NLE T3SS effector | NLE mediated host cell effects |
|  | 0532 | Q58R | 3 & 4 | RTX-Family Toxin | Host-cell damage |
| pO157 | *hlyA* | E157A | 4 | Hemolysin | Host-cell damage |
|  | *hlyC* | L208I | 4 | Hemolysin | Host-cell damage |
| Adherence |  |  |  |  |  |
| Chromosome | *cah* | G779W | 5 | Calcium-binding antigen-43 homologue | Non-fimbrial adhesin |
|  |  | L781A | 5 |  |  |
|  |  | E782R | 5 |  |  |
|  |  | T783Opal | 5 |  |  |
|  | *csgG* | P76T | 3 & 4 | Curli production assembly component | Usher protein |
|  | *eaeH* | T1301K | 3 & 4 | Putative adhesin with Ig-like class 3 domain | Non-fimbrial adhesin |
|  | *efa1'* | T285P | 1 & 3 & 4 | Central fragment of *efa1* | Non-fimbrial adhesin |
|  | *fimA* | V100A | 4 | Major type 1 subunit | Fimbrial-like adhesion |
|  | *fimB* | S80P | 2 | Tyrosine recombinase | Regulator |
|  | *fimH* | K156N | 3 & 4 | Minor fimbrial subunit | Fimbrial-like adhesion |
|  | *fmlC* | D532G | 4 | F9 fimbriae usher | Usher Protein |
|  | *htrE* | H344Q | 1 | Putative fimbrial usher | Usher Protein |
|  |  | T460A | 3 |  |  |
|  | *ppdB* | P158L | 3 & 4 | Prepillin peptidase | Chaperone |
|  | *wzzB* | P136S | 3 & 4 | O-antigen short chain regulator | Regulator |
|  | *ybgO* | V46A | 3 & 4 | Predicted fimbrial-like adhesin | Fimbrial-like adhesion |
|  | *ycbF* | R69S | 3 & 4 | Predicted periplasmic pilin chaperone | Chaperone |
|  | *yehC* | A32V | 3 | Predicted periplasmic pilin chaperone | Chaperone |
|  | *yfaL* | S23P | 3 & 4 | Type V AIDA-like adhesin | Non-fimbrial adhesion |
|  |  | D38N | 3 & 4 |  |  |
|  |  | T104I | 3 & 4 |  |  |
|  | *yfcS* | P163H | 5 | Predicted periplasmic pilus chaperone | Chaperone |
|  | *yfcU* | G746A | 3 & 4 | Putative fimbrial usher | Usher Protein |
|  | 0341 | R218Q | 3 & 4 | Putative adhesin | Non-fimbrial adhesion |
|  | 2924 | I282K | 5 | Putative fimbrial-like adhesin | Fimbrial-like adhesion |
| pO157 | *toxB* | V237D | 4 | Cytotoxin B | Non-fimbrial adhesion |
|  |  | T1888I | 4 |  |  |
| Virulence-associated | |  |  |  |  |
| Chromosome | *chuA* | G259E | 3 & 4 | Outer membrane heme receptor | Nutrient acquisition |
|  |  | I577N | 5 |  |  |
|  | *chuS* | K11T | 5 | Heme oxygenase | Nutrient acquisition |
|  | *phoB* | Q179L | 3 & 4 | Phosphorus regulator | Signaling |
|  | *terD* | M155R | 1 & 2 & 4 | Tellurite resistance | Resistance |
|  | *terF* | Opal103S | 4 | Tellurite resistance | Resistance |
|  | *wcaJ* | D216D | 4 | Colanic acid lipid carrier transferase | Biofilm/capsule biogenesis |
|  | *wcaL* | R73S | 3 & 4 | Colanic acid glycosyl transferase | Biofilm/capsule biogenesis |
|  | *wcaM* | L29F | 3 & 4 | Predicted colanic acid biosynthesis protein | Biofilm/capsule biogenesis |

* Reference with SNP: 1 - EC4115; 2 - TW14359; 3 - EDL933; 4 - Sakai; 5 - All four strains
